# Insight into Extracellular Vesicle-Cell Communication: From Cell Recognition to Intracellular Fate

**DOI:** 10.3390/cells11091375

**Published:** 2022-04-19

**Authors:** Lana Ginini, Salem Billan, Eran Fridman, Ziv Gil

**Affiliations:** 1 Rappaport Family Institute for Research in the Medical Sciences, Technion–Israel Institute of Technology, Haifa 31096, Israel; lana.ginene@gmail.com (L.G.); eran.frid@gmail.com (E.F.); 2 Head and Neck Institute, The Holy Family Hospital Nazareth, Nazareth 1641100, Israel; billan.s49@gmail.com; 3 Medical Oncology and Radiation Therapy Program, Oncology Section, Rambam Health Care Campus, HaAliya HaShniya Street 8, Haifa 3109601, Israel

**Keywords:** extracellular vesicles (EVs), exosomes, endocytosis, lipids, proteins

## Abstract

Extracellular vesicles (EVs) are heterogamous lipid bilayer-enclosed membranous structures secreted by cells. They are comprised of apoptotic bodies, microvesicles, and exosomes, and carry a range of nucleic acids and proteins that are necessary for cell-to-cell communication via interaction on the cells surface. They initiate intracellular signaling pathways or the transference of cargo molecules, which elicit pleiotropic responses in recipient cells in physiological processes, as well as pathological processes, such as cancer. It is therefore important to understand the molecular means by which EVs are taken up into cells. Accordingly, this review summarizes the underlying mechanisms involved in EV targeting and uptake. The primary method of entry by EVs appears to be endocytosis, where clathrin-mediated, caveolae-dependent, macropinocytotic, phagocytotic, and lipid raft-mediated uptake have been variously described as being prevalent. EV uptake mechanisms may depend on proteins and lipids found on the surfaces of both vesicles and target cells. As EVs have been shown to contribute to cancer growth and progression, further exploration and targeting of the gateways utilized by EVs to internalize into tumor cells may assist in the prevention or deceleration of cancer pathogenesis.

## 1. Introduction

Intercellular communication can occur via a wide variety of signaling processes, and is indispensable for the maintenance of homeostasis [1]. One of these processes is the cellular secretion and uptake of extracellular vesicles (EVs) [2]. These are lipid bilayer-enclosed membranous structures secreted by cells under physiological conditions, or as a reaction to specific stimuli [3].

Extracellular vesicles comprise heterogeneous populations of various sizes, and subcellular origin of membranes. Although the classification of EVs is continually evolving [4,5,6], they generally fall into three major categories [2,7]: (1) apoptotic bodies—whose size ranges from 50 nm to 5 μm, which are released when plasma membrane blebbing occurs during apoptosis and are therefore excluded from this review; (2) microvesicles—whose size ranges from 50 nm to 1 μm, which directly bud from plasma membranes; and (3) exosomes, the smallest EVs—whose size ranges from 30 to 150 nm in diameter and have endocytic origins. In this review, we shall focus on exosomes.

To be clear, EV nomenclature has yet to be established. Therefore, discrimination of EV subsets remains somewhat arbitrary. Despite the variety of nomenclatures that have been used to describe small EVs over the years, for the sake of clarity and according to the protocols the authors adopted for EV isolation, this article will refer to small EVs as exosomes.

Under the scanning microscope, exosomes present with a characteristic cup-shaped morphology [8], and are formed as intraluminal vesicles (ILVs) in multivesicular bodies (MVBs) through inward invagination of the endosome membrane. MVBs subsequently fuse with the plasma membrane and release their sequestered ILV contents as exosomes into the extracellular environment [9]. A well-understood mechanism for the formation of ILVs is through the recruitment of the endosomal sorting complex required for transport (ESCRT) machinery to ubiquitinated endocytosed cargoes [4,10]. Alternative cargo recruitment and ILV budding mechanisms have been described as involving lipid organization, including the generation of ceramide to induce membrane curvature [11]. Generally, exosomes are released by all types of eukaryoṭic cells and are widely available in almost all bodily fluids, including breast milk, cerebrospinal fluid, saliva, peripheral blood, ascites, and urine [12].

Exosomes are carriers of preassembled complex biological information, which are necessary for cell-to-cell communication by transferring cargo molecules that elicit pleiotropic responses in recipient cells in physiological and pathological processes [13,14]. This intercellular trafficking can also occur with neighboring cells as a form of paracrine signaling and/or with distant cells as a form of endocrine signaling [15,16].

Accumulating evidence suggests that exosomes play essential roles in the regulation of molecular pathways in malignancies and in remodeling the tumor immune microenvironment, and are critically responsible for the migration and proliferation of tumor cells, as well as the angiogenesis, drug resistance, and metastasis of said cells [17,18]. The interplay between cancer cells, via the exchange of exosomes, may promote the transfer of oncogenes and onco-miRNAs (oncomirs) from one cell to another, thus leading to the reprogramming of the recipient cell and enhancing prometastatic behavior [19,20,21]. Regardless, it has already been established that cancer cells exchange exosomes with stromal cells to create a protumor microenvironment, as well as to increase tumor invasion and proliferation [22,23]. Newly published research suggests that several subpopulations of EVs demonstrate differing abilities to activate fibroblasts into a cancer-associated fibroblast (CAF)-like state, supporting their proliferation, motility, invasiveness, and enzyme expression. Additionally, in some activation processes, exosome-like EV subpopulations seem to be more efficient than exosome-like and microvesicle-like EVs [24]. A further study ascertained that cancer cells facilitate oncogenesis via exosomes and ectosomes secreting oncogenic cargo into the tumor microenvironment, and revealed that, in comparison to ectosomes, exosomes induced significant cell proliferation and migration of the recipient cells [25]. Additionally, tumor-derived exosomes can reshape distant microenvironments, such as pre-metastatic niches, driving organ-specific metastasis and thus, for example, by pre-conditioning the brain microenvironment with exosomes derived from brain metastatic cells, a metastatic niche is created that supports colonization through the induction of endothelial cell branching and inflammation in the perivascular niche [26].

A number of research studies have offered evidence supporting the potential role of tumor-derived exosomes in angiogenesis. Indeed, a recent investigation showed that exosomes derived from 5-fluorouracil-resistant colon cancer cells contain high levels of GDF15, thereby enhancing angiogenesis by inhibiting the Smad signaling pathway [27]. Other studies have demonstrated that treatment with exosomes obtained from breast cancer cells enhanced the expression of VEGF-A in human umbilical vein endothelial cells, and elevated the capacity of those endothelial cells to migrate and generate vessel-like structures [28]. Contemporary evidence points toward tumor-derived exosomes playing a central role in modulating both the innate and adaptive immune systems, thus influencing pathophysiological processes [29]. Recent papers showed that glioma-derived exosomes and Lewis lung carcinoma-derived exosomes were implicated in M2 macrophage differentiation, which subsequently promoted cancer proliferation, migration, and invasion [30,31].

In addition to tumor-derived exosomes, stromal cell-derived exosomes are also important contributors to tumor microenvironment modulation. The transmission of miR-365 via M2 macrophage-derived exosomes significantly decreased the sensitivity of pancreatic cells to gemcitabine, in vitro and in vivo. Moreover, mice deficient of Rab27 a/b genes, which lack exosomal secretion, responded significantly better to gemcitabine than did wild-type mice. These results demonstrate that macrophage-derived exosomes act as key regulators of gemcitabine resistance in pancreatic ductal adenocarcinoma [32]. Similarly, m2 macrophage-derived exosomes display a high expression level of miR-21-5p and miR-155-5p. These microRNA (miRNA) are responsible for the migration and invasion of colorectal cancer cells [33].

As major producers of extracellular matrix (ECM), fibroblasts are involved in regulating tissue remodeling and repair, and fibroblast-derived exosomes may act in tumor cells, as well as in other non-tumor cells of the tumor microenvironment [17]. In pancreatic cancer, fibroblasts exposed to gemcitabine significantly increase the release of exosomes. These exosomes increase the chemoresistance-inducing factor, Snail, in pancreatic cancer cells and promote proliferation and drug resistance [34]. In addition, exosomes derived from CAFs have been shown to provide certain nutrients to pancreatic and prostate cancer cells, and to drive them to glycolysis. Zhao et al. demonstrated that fibroblast-derived exosomes reprogram cancer cell metabolism by increasing glucose uptake, lactate secretion, and reducing the oxygen consumption rate, providing de novo “off the shelf” metabolites through exosomal cargo [35].

Differences evident in exosomal composition offer indications of the current state of the secreting cell [12,16]. This particular characteristic of exosomes can be used as a potent screening tool for cancer prediction; thus, tumor-derived exosomes hold great promise as a liquid biopsy tool for cancer diagnosis and prognosis [36]. In particular, miRNAs are found inside exosomes, as are long non-coding RNA (lncRNA) and circular RNA, which can easily be extracted via a minimally invasive procedure, such as blood or serum samples [16,37,38]. Various studies have been performed to characterize exosomal miRNA as diagnostic biomarkers for cancer, which have provided evidence that several exosomal miRNAs—miR-620 [39], miR-126 [40] and other miRNAs (hsa-miR-9-3p, hsa-miR-205-5p, hsa-miR-210-5p, and hsa-miR-1269a) [41]—can be used as early diagnosis and prognostic markers for lung cancer.

It has also been demonstrated that there are many promising circulating miRNA biomarkers for pancreatic cancer—a deadly malignancy that is all too often diagnosed at later stages. Notable here are the elevated expression levels in pancreatic neoplasm of serum exosomal miR-191, -21, -451a, which are considered to be efficient diagnostic markers [42]. Furthermore, the exosomal miRNA panel containing miRNA-122, miRNA-21, and miRNA-96 could be defined as a diagnostic biomarker for patients with hepatocellular carcinoma [43]. New data indicate that the circulating lncRNA-colon cancer-associated transcript 2, which may be protected by exosomes, can serve as a novel potential predictor in colon cancer [44]. Additional research revealed that some unique exosomal proteins were associated with the existence of tumor cells, thus facilitating early cancer detection and prognosis prediction [45]. For example, the exosomal αvβ3 integrin may be clinically useful as a non-invasive biomarker and diagnostic tool to follow prostate cancer progression [46].

As there is also profound potential for exosomes to serve as endogenous and versatile carriers for the delivery of various therapeutics; significant efforts are being devoted to explore this possibility. In contrast to liposomes, injected exosomes can efficiently enter other cells and deliver functional cargos because they carry various surface proteins. Furthermore, exosomes can effectively avoid phagocyte cells and penetrate biological barriers (e.g., blood-brain barrier) and the ECM, thus allowing them to be widely distributed and stable in biofluids. In addition, researchers are encouraged about the therapeutic application of exosomes since they have already been demonstrated to have better biocompatibility and lower immunogenicity compared to synthetic nanodelivery systems [6,47,48].

In many studies, exosomes have been used as delivery vectors for small-molecule and chemotherapeutic agents. It has been reported that, in comparison to liposomes, exosomes engineered to carry short interfering RNA or short hairpin RNA specific to oncogenic KrasG12D, a common mutation in pancreatic cancer, exerted superior tumor growth suppression and inhibited metastasis [49]. Trastuzumab emtansine (T-DM1), an antibody-drug conjugate that carries a cytotoxic drug (DM1) bound to HER2-positive cell-derived exosomes, has been shown to accumulate in HER2-positive SKBR-3 cells, resulting in viability reduction and activation of caspases 3 and/or 7 [50].

Recently, Zhan et al. improved the interaction efficiency between reticulocyte (RTC)-derived exosomes with tumor cells by regulating their lipid composition with phosphatidylcholine (PC) to achieve better intracellular delivery of therapeutic agents. They showed that PC modification significantly enhanced (by approximately 51%) the cellular uptake of exosomes in human breast cancer cells. Moreover, a quantitative result suggested that the intracellular fluorescence intensity of human glioblastoma cells treated with PC-exosome-doxorubicin (Dox) was nearly 2.4-fold stronger than that of the exosome-Dox group [51]. In mice bearing human lung A549 tumor xenografts, oral administration of exosomal paclitaxel exerted significantly pronounced tumor suppression compared to those receiving free paclitaxel intraperitoneally [52].

A great deal has been learned about EV biogenesis, the cargo that they contain, and the biological effects that they promote in normal physiological processes and in disease, in addition to their therapeutic potential. However, it is important to have a clear understanding of the molecular mechanisms involved in EV targeting and attachment, as well as the processes by which they are internalized by recipient cells. In this review, we will highlight current knowledge regarding how EVs enter target cells.

## 2. Cellular Recognition of EVs

### 2.1. Selective Uptake

The rising interest in EVs is linked to their capacity to be selectively taken up by cells and to trigger phenotypic changes in acceptor cells as a result of the molecular cargo they carry. Extracellular vesicles derived from different sources have been reported to interact preferentially with specific cell types [53,54,55]. For example, tumor microenviromental cytokines, particularly CCL2, decorate cancer exosomes and thereby alter their systematic biodistribution such that they are selectively taken up by cytokine receptor-positive cells in specific tissues, which results in immune system modulation of pre-metastatic niche and subsequent increase in metastatic burden [56]. In another in vivo study, Pable et. al. were able to detect the preferential accumulation of melanoma exosomes in small metastatic tumors located in the lungs when compared with other organs [57].

Of note, a recipient cell can also be the producing cell itself and thereby generate autocrine responses [58,59]. There is also increasing evidence that the specificity of EV uptake depends on cellular origin. Using hollow gold nanoparticle (HGN)-loaded exosomes, researchers were able to follow cell–cell communication and, in particular, how exosome cellular origin can affect the transference of HGNs between cells. In that study, preferential transfer was exploited to selectively induce death by hyperthermia only in the cell of origin [60].

Additional evidence of selective uptake was reported by Seung et al., who reported that as compared to epithelial cell-derived exosomes, cancer-derived exosomes provide potential vehicles for effective in vivo delivery via selective accumulation in ovarian cancer tumors of SKOV3 xenograft mice, most likely because of their cell tropism [61]. In in vitro and in vivo studies, autologous colorectal cancer cell (C26) exosomes were more efficiently taken up by C26 cancer cells when compared to allogeneic melanoma (B16BL6) exosomes [62]. It is notable that the interaction and uptake of EVs by recipient cells may be dependent on the specific properties of recipient cells [63,64].

The preferential interactions between EVs and certain cell types have also been observed not only in cancer, but in other pathophysiological conditions [65,66]. In the case of traumatic brain injury, intracerebroventricular microinjection of human adipose mesenchymal stem cell (hADSC)-derived exosomes (hADSC-ex) specifically enter microglia/macrophages and suppress their activation during brain injury, thereby inhibiting inflammation and facilitating functional recovery [67]. Non-pigmented ciliary epithelium (NPCE)-derived exosomes could be easily identified in trabecular meshwork cells. In all of the other cell lines investigated, including ocular (RPE), epithelial (MCF7, SKOV-3, LnCap), and endothelial (CEND) cells, only a few NPCE-derived exosomes were detected [68].

The mechanisms involved in the preferential uptake of EVs are not yet totally understood. However, there is evidence that differences in EV surface components probably influence their recognition and capture by target cells. Target cell specificity is likely to be determined by specific interactions between EV surface components and receptors at the plasma membrane of the recipient cells. Much of this specificity can be explained by protein surface and lipids.

### 2.2. Proteins

The recognition of, and binding between exosomes and target cells, has been reported to involve proteins present on the cell surface of both exosomes and target cells that facilitate subsequent internalization (Supplementary Table S1). Many of these interactions have been elucidated by using Proteinase K (PK) and other enzymes for the deglycolization of proteins [69,70], or by the use of specific antibodies or peptides, resulting in a steric block that prevents their interaction [71,72,73]. Internalization of thymic exosomes by thymic CD4+ thymocytes was completely inhibited by pre-treatment of thymic exosomes with PK, whereas no reduction was seen for thymic dendritic cells, which strongly supports the role of proteins in the exosomal uptake pathway [74].

#### 2.2.1. Tetraspanin

The transmembrane 4 superfamily (TM4SF), or tetraspanins, are small transmembrane proteins expressed in many species. Tetraspanins are implicated in a diverse range of biological processes, including physiological cell adhesion, motility, activation, and proliferation, in addition to pathological cancer metastasis and viral infection [75,76]. Exosomal formation and secretion from MVBs were initially believed to be dependent on ESCRT. However, knockdown studies have established that MVBs can be formed in the absence of all four ESCRT proteins, and that exosomal formation can also be modulated by other proteins, such as tetraspanin. Different tetraspanin members that have been shown to regulate cargo sorting into exosomes could influence the selectivity of exosomal assembly [77,78]. Tetraspanins are highly abundant on the surface of exosomes and CD63, CD9, and CD81 are well-established exosomal markers. Moreover, increasing evidence is emerging that tetraspanins do not only function at the level of EV biogenesis, but may also play direct or indirect roles in EV-mediated cargo transfer or bioactivity in recipient cells [79,80,81]. For example, Rana and Zöller have demonstrated that the integrin/tetraspanin expression pattern found in exosomes can influence their targeting [53].

Some studies have highlighted the role of CD9 in exosomal docking and uptake into recipient cells [82,83,84]. In a study conducted in 2019, human perivascular stem cell-derived exosomes were observed to promote bone marrow mesenchymal stem cell migration, proliferation, and osteogenic differentiation. These effects on the recipient cells were dependent on exosomal surface-associated tetraspanins along with their known binding partners, such as immunoglobulin superfamily member 8 (IGSF8) and prostaglandin F2 receptor inhibitor (PTGFRN), as demonstrated by using neutralizing antibodies for CD9/CD81 [82]. Similar results were obtained in new research which shows that the anti-CD9 neutralizing antibody abrogated the uptake of exosomes from fibroblast cells (CaF64) in both scirrhous-type gastric cancer cell lines, NUGC-3 cells, and OCUM-12 cells [83]. Tetraspanins on the surface of recipient cells are also involved in exosomal binding. Masaharau et al. have suggested that radiation exposure increased CD81 tetraspanin-enriched microdomain association with CD29 on the surface of mesenchymal stem cells (MSCs). The radiation also led to preferential attachment of the exosomes to the complex by modifying their molecules, or via another unknown factor. Thus, they concluded that CD81, cooperating with CD29, plays a central role in the radiation-induced increase in the uptake of exosomes in MSCs [85].

Other important players in exosomal binding and attachment are CD63 [86], TSPAN8, and CD151 [87,88]. While fluorescence-activated cell flow cytometry and immunocytochemistry staining studies have indicated that exosomes significantly increase the uptake of fluorescence-labeled siRNA in autologous brain endothelial cells, decreased fluorescence intensity was observed in those cells that were treated with CD63-blocked exosomes, which thereby blocked exosome-delivered siRNA. Thus, brain endothelial cell-derived exosomes could potentially be used as a natural carrier for brain delivery of exogenous siRNA in the treatment of brain tumors [86]. Using rat pancreatic adenocarcinoma CD151/TSPAN8 knockdown exosomes had little or no effect on stroma cell activation—upregulation of cytokines, cytokine receptors, and proteases—and the promotion of inflammatory cytokine expression in hematopoietic cells, all of which are at least partly due to reduced binding/uptake of CD151-deficient and/or Tspan8-deficient exosomes [88].

#### 2.2.2. Integrin

The integrin family consists of heterodimeric α- and β-subunits, each of which is a type I transmembrane receptor. Integrins are primarily responsible for mediating cell and ECM attachments, and are also involved in cell-cell interactions, which are essential for cell adhesion and spreading, migration, ECM organization, and coupling the extracellular environment to intracellular signals. Integrin adhesion complexes have been linked to components of the intracellular trafficking machinery, and accumulating data now reveal that they are key regulators of endocytosis and exocytosis [89,90]. Another emerging role for integrins in vesicle traffic relates to the binding and uptake of EVs [91,92,93].

Treating leukemia-derived exosomes with antibodies to inhibit exosome integrin αV and β3 significantly diminished the association of leukemia-derived exosomes to human choroid plexus papilloma cells [94]. Similarly, pre-treatment of the exosomes of human primary astrocytes with RGD peptides (an integrin ligand) significantly prevented exosomal uptake by neurons, thus demonstrating the significant contribution of integrins [95].

There are also suggestions that some EVs contain integrins that can mediate the docking of EVs onto certain cell types/organs. This has been demonstrated in the context of cancer metastasis, wherein the uptake of EVs in those cell types/organs creates a pre-metastatic niche which favored the growth of metastases. For instance, an in vitro study showed that integrin α2β1 mediated CAF-exosome uptake by lung fibroblasts. Meanwhile, in an in vivo study, CAF-exosomes injected into the tail veins of mice were observed principally in the lungs, and blockage by TC-I 15 (an α2β1 integrin inhibitor) suppressed the uptake of CAF-exosomes by lung fibroblasts, preventing pre-metastatic niche formation and subsequent metastasis in the lungs [96]. Furthermore, exosome proteomics have revealed that integrin expression profiles correlated with tissue organotropism, specifically that breast cancer cell exosomes expressing ITGα6β4 and ITGα6β1 colocalized with stromal cells in a laminin-rich lung microenvironment; furthermore, ITGαvβ5-expressing pancreatic exosomes colocalized with macrophages in a fibronectin-rich liver [97].

Another study showed that perturbation of α3β1 integrin on ovarian cancer cell-derived exosomes leads to an attenuation of exosomal uptake into their parent cells [98]. The interactions between integrin LFA-1 and ICAM-1 had previously been implicated in the uptake of macrophage exosomes in brain microvascular endothelial cells (BMECs) comprising the blood–brain barrier (BBB) [73]. Tested in a recent study, exosomes of different origins—variably expressed CD46, AVβ6, AVβ3, and ICAM-1—readily crossed the BBB, suggesting that this might be regulated in part by differences in integrin expression levels on exosomal surfaces [99].

Integrin-mediated EV-cell interactions can also occur reciprocally, as exemplified in Refs. [59,72,100,101,102,103,104,105]. The principal finding of a newly published study revealed that a major component of hepatocyte exosomes, fibronectin, mediates exosomal binding to integrin on the target cells, and favors exosomal uptake by endocytic mechanisms [100]. The expression of laminin γ2, an integrin ligand, on the surface of exosomes derived from metastatic oral squamous carcinoma cells mediated the uptake of these exosomes by lymphatic endothelial cells. This uptake has been shown to be dependent on the cellular expression of integrin α3, while knockdown of exosome-expressed laminin γ2 decreased exosomal drainage into lymph nodes after intra-tumoral administration [102].

In a similar fashion, specific uptake of exosomes derived from gastric epithelial cells into the cytoplasm of gastric cells depends on integrin α6 and αX. When gastric cancer cells AGS and MKN1 were treated with gastric-derived exosomes, these exosomes were clearly localized in the cytoplasm of those cells. However, knockdown of integrin α6 and αX in the recipient cells markedly inhibited the internalization of exosomes into the cytoplasm of those cells [59].

#### 2.2.3. Proteoglycans

Proteoglycans (PGs) are complex macromolecules which are composed of a core protein covalently decorated with linear glycosaminoglycan (GAG) chains—including alternating glucuronic acid and N-acetylglucosamine or N-acetylgalactosamine residues—that give rise to heparan sulfate PGs (HSPGs) and chondroitin sulfate PGs (CSPGs), respectively [106]. PGs orchestrate key steps of cancer cell invasion and metastasis, as well as associated cell reprogramming [107]. Additionally, HSPGs have been identified as key players in exosomal biogenesis and uptake [108]. In a current study, it was shown that syntenin not only supports the endosomal budding of cargo and exosome production, but also controls the uptake of exosomes and the effectiveness of viral transductions. Syntenin controls the expression levels of syndecans and CD63, both of which are involved in loading exosomes with cargo in exosome-producing cells and retrieving cargo from exosomes [109].

Multiple studies have shown that PGs mediate EV binding to targeted cells [101,110,111,112,113,114,115,116]. New data demonstrate that exosomes derived from neural stem cells (NSCs) efficiently carry protein cargo across the BBB by using HSPGs on endothelial cells, which act as receptors. This suggests promise for the improvement in drug delivery to the brain by targeting nanomedicine to HSPGs [110]. Indeed, in a recently published paper, strategies cited include competitive inhibition of HSPG by the HS mimetic heparin; digestion of cell surface HSPG by specific HS lyases; pharmacologic inhibition of HSPG biosynthesis by the false substrate 4-nitrophenyl β-D-xylopyranoside; and inhibition of HS sulfation and polyanionic charges via NaClO_3_ treatment. Interestingly, regardless of the strategy used to interfere with HSPG function, researchers consistently found inhibition of exosomal uptake in glioma cells [111]. In a similar fashion, PGs expressed on breast cancer-derived exosomes may bind other molecules, such as cytokines (CCL2), causing exosomal accumulation in specific cell subsets and organs [56].

One study provided insights into the molecular mechanism mediating the interaction and internalization of Trichomonas vaginalis exosomes by host cells. The researchers showed that exosomes express the 4-α-glucanotransferase, a carbohydrate-binding protein which they defined as exosomal ligands, and found them to bind HSPGs on mammalian cells as a means of mediating exosomal uptake [117]. Additionally, histones were also reported to function as ligands for the uptake of exosomes by tumor cells via syndecan-4 or other HSPGs [118,119]. Of note, studies have shown that fibronectin tethers to HSPGs on the exosome, which suggests that this could act as a scaffold for exosomal docking to HSPGs lying on the surface of normal and tumor cells [120,121].

#### 2.2.4. Glycans and Lectins

##### Glycans

Carbohydrate structures are conjugated to lipids and proteins as glycans or as repeating glycosaminoglycan chains in proteoglycans, and these exert different functions on different size scales [122,123]. The unique presence of certain EV surface glycans holds the potential for these to be targetable for the development of novel EV detection and isolation methodologies. For example, approaches targeting cancer-derived EVs using glycan-specific moieties might effectively be used in cancer diagnosis and detection [124,125,126]. Moreover, the role and impact of glycosylation in EV biodistribution, uptake and protein cargo sorting have already been proven [127,128,129]. Additionally, lectin/glycan interactions have been associated with recognition and uptake of glioblastoma-derived exosomes by dendritic cells [130].

Alterations at the exosome terminal N-glycan with Peptide-N-Glycosidase F (PNGase F) have been described as resulting in enhanced cellular uptake by peritoneal macrophages. Despite the effect of PNGase F on cellular uptake, deglycosylation had minimal impact on the in vivo pharmacokinetics of exosomes, which showed almost identical clearance to those of untreated exosomes [131]. It has been speculated that the increase in affinity for exosomes after treatment with glycosidases is attributed to the fact that modification of glycan structures removes steric hindrance of other vesicle surface ligands, which are then able to encounter their cell surface receptors [132].

In line with these results, the suppressive effect of N-glycans and O-glycans was also observed in breast cancer-derived EV uptake into human umbilical vein endothelial cells in vitro. However, N-deprived EVs did not alter biodistribution in mice, and O-linked glycan removal resulted in lung accumulation [133]. The authors suggest that surface glycosylation controls EV delivery by inhibiting non-specific binding to proximal tissues, thus enabling other surface proteins to target destination organs. In contrast to the previously referenced papers, glycoamidase PNGase F drastically remodeled the surface oligosaccharides and blocked the uptake of helminth parasite-secreted EVs by host macrophages [134].

It has also been demonstrated that removal of the terminal sialylation through treatment with neuraminidase (NA)—a glycosidase that cleaves terminal sialic acid residues from glycoproteins, glycopeptides, and oligosaccharides—also led to a non-significant increase in exosomal uptake by ovarian cancer cells [70]. However, removal of sialic acid modifications via NA treatment increased the amount of prostate cancer exosomes internalized by macrophage cells [135]. Additionally, treated exosomes isolated from mouse liver progenitor cells with neuraminidase showed an increased accumulation of treated exosomes in the lungs and axillary lymph nodes when compared to non-treated exosomes [136]. The enzyme activity on EV sialic acid changes the dynamic biological behavior of the EVs by reducing the negative charge toward neutrality. Correspondingly, less electrostatic repulsion occurs between these exosomes and negatively charged cell membranes, and steric hindrance of their sialic acid is decreased [132,137].

##### Lectins

Lectins are proteins whose main function is to recognize and bind to specific glycan moieties. There are three classes of lectins—transmembrane, calcium-dependent, C-type lectins, and selectins (selectin-E, -P, and -L); transmembrane, sialic acid-binding lectins, termed siglecs; and cytosolic and secreted beta-galactoside-binding S-type lectins, termed galectins [127,138]. In humans, one hundred sixty (160) putative lectins have been identified and implicated in various cellular functions, including protein folding, intracellular trafficking, cell adhesion, and pathogen recognition [139,140,141].

Emerging evidence suggests that lectins are enriched in EVs and likely contribute to their ability to attach to recipient cells [73,128,142,143,144,145]. Asako et al. have shown that exosomes collected from MSCs uptake involved recognition by HeLa cell surface-bound sialic acid-binding immunoglobulin (Ig)-like lectins (siglecs), as antibody blocking and competition with sialic acid decrease uptake of MSC-exosomes. Confirming this fact regarding siglec-related uptake, in vivo experiment results have shown that exosomes were specifically taken up into siglec-positive antigen-presenting cells [128].

The accumulation of macrophage-derived exosomes in BMECs was decreased by a panel of carbohydrates and the calcium chelator, ethylene glycol-bis (2-aminoethylether) -N, N, N′, N′-tetraacetic acid (EGTA), along with DEC205 antibodies [73]. Human breast milk exosomes—which carry MUC1 on their surface, a known DC-SIGN ligand-bind to DC-SIGN (C-type lectin receptor) on monocyte-derived dendritic cells [144]—have been found to act as a novel protective factor against the vertical transmission of HIV-1 by competing with the virus. Additionally, it has been determined that sialoadhesin CD169 (Siglec-1) is required for the capture of B cell-derived exosomes into macrophages via their surface-expressed α2,3-linked sialic acids [145].

Lectins were also reported to be involved in intracellular signaling, in that dengue virus-activated platelets were found to secrete exosomes that bind and activate CLEC5A (a C-type lectin) on neutrophils, thus further enhancing neutrophil extracellular trap formation [146]. However, lectin can compromise EV functionality. For example, interactions between glycoproteins and soluble lectins released into the intercellular compartment can play a prominent role in the fate of EVs. Specifically, in a 2017 study it was shown that REG3β, a soluble C-type lectin, binds the surface of macrophage-derived exosomes through its lectin domain, thereby interfering with internalization into pancreatic cancer cells [147].

### 2.3. Lipids

Several works have extensively reported on the lipidic composition of exosomes derived from different sources, and the enrichment of some lipid classes of the exosomal membrane as compared to their producer cells. Exosome membranes display a particular lipid organization and composition, and have been shown to be enriched with cholesterol (CHol), diglycerides, sphingolipids (e.g., sphingomyelin and ceramide), phospholipids, glycerophospholipids (e.g., phosphatidylcholine (PC), phosphatidylserine (PS), phosphatidylethanolamine (PE), and phosphatidylinositol (PI)), and 6-polyglycerophospholipids. Curiously, however, the content of lipids in exosomes differs substantially from that of the parental cells [148,149]. Several studies have shown two to three times enrichment from cells to exosomes of Chol, PS, and sphingolipids, particularly sphingomyelin. In contrast, exosomes generally contained less PC and PI (mol% of total lipids) than their parent cells, and only small changes were reported for PE in most studies [150,151,152]. Currently, multiple mechanisms involved in exosome biogenesis have been identified. However, some research has indicated the existence of ESCRT-independent mechanisms for ILV formation and release, where one of them is the denominated lipid-driven mechanism [10].

In addition to proteins, EV lipids appear to be involved both in the internalization and fate of exosomal material into recipient cells. In the context of EV-cell interactions, the material most examined has been PS [63,153,154,155,156,157,158,159,160,161]. The infectivity of exosomes derived from hepatitis A virus (HAV)-infected cells (exo-HAVs) was blocked by liposomes containing PS:PC:Chol and PS:PC, but not PC:Chol and PC, which suggests a shared mechanism for the uptake of liposomes and exo-HAVs [153]. Using a similar methodology, negatively charged PS-liposomes suppressed the cellular uptake of melanoma-derived exosomes by macrophages [155]. In addition, T cell immunoglobulin and mucin receptor 1 (TIM-1), a PS receptor, was shown to be a functional receptor for the entry of exosomes into recipient cells [154]. Exosomal lysoPC could play a vital role in exosome-cell interactions, as it has been reported that it attracts T lymphocytes to lymph nodes and allows exosomes to interact with apoptotic cells, resulting in the subsequent elimination of them both [162,163].

Recently, Anil et al. have suggested that the uptake of intestinal epithelial cell-derived exosomes by specific liver cell types is dependent on their lipid composition, specifically the percentage of PC lipids they contain, thus leading to inhibition of the insulin signaling pathway. Further, they revealed that higher PC concentrations on exosomes result in more exosomes being taken up by macrophages, and thereby providing a strategy for engineering exosomes to become targeting delivery vehicles for a variety of molecules [164]. In an attempt to improve the tumor-specific bioavailability of exosomes, PC molecules were inserted into the membranes of reticulocyte-derived exosomes to generate PC-exosomes. The results demonstrated that PC-exosomes had enhanced tumor cell uptake and exhibited a higher efficiency in delivering small molecular drugs into cancer cells, thereby enhancing tumor cell growth inhibition [51].

Cholesterol, PE, and cardiolipin could also play vital roles in exosome-cell interactions [165,166,167,168,169]. For example, cholesterol in cellular membranes has been shown to contribute to exosomal membrane fusion, as fusion was inhibited by the cholesterol-binding compound filipin [165].

## 3. Extracellular Vesicle Uptake Mechanism

**Figure 1:**
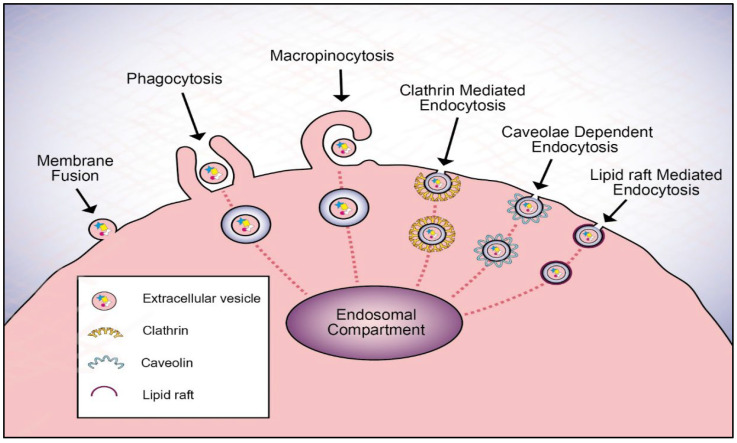
Extracellular vesicle (EV) internalization pathways into recipient cells. Exogenous EVs have been shown to be internalized by cells via multiple endocytic pathways depending on cell type. These pathways include the following: 1-phagocytosis, a process used by professional phagocytic cells that involves the engulfment and digestion of extracellular material into plasma membrane-derived vacuoles called phagosomes; 2-macropinocytosis, a process during which the cell extends large membrane ruffles that fold back onto the cell surface and fuse to form pockets; 3-clathrin-mediated endocytosis, a process that requires interactions between ligands on the EV surface and specific receptors present on the plasma membrane which subsequently results in EV-packed clathrin-coated vesicles; 4-caveolae-dependent endocytosis, a process that is characterized by flask/cave-like invaginations marked by the presence of caveolin-1 and 5-lipid raft-mediated endocytosis, which takes place in a cholesterol- and sphingolipid-enriched microdomain that is regulated by various molecules. Internalization via different endocytotic pathways will target exogenous EVs in the endosomal compartment. EVs may also enter cells via fusion with the plasma membrane to deliver their contents.

Depending upon the cell type, EVs communicate their messages or deliver cargo to recipient cells in different ways. First, once EVs attach to a cell, they can remain bound to the surface and can activate receptors expressed on target cells, thus initiating intracellular signaling pathways without being internalized. Second, EVs can transfer their surface proteins and cytoplasm to target cells through subsequent fusion with the plasma membranes of target cells [170,171]. Third, EVs may also be internalized by endocytosis. Some of the widely discussed mechanisms for EV endocytosis uptake are phagocytosis, macropinocytosis, clathrin-mediated endocytosis (CME), caveolae-dependent endocytosis (CDE), and lipid raft-mediated endocytosis (Figure 1) [172]. Notably, to exert its functional activity, some EV cargo must be released from the EV after the vesicle breaks and bursts. As Giulia et al. demonstrated, despite a greater amount of VEGF in vesicles of the overexpressing human ovarian carcinoma, they had the same chemotactic activity as low-VEGF vesicles from control cells, suggesting that VEGF was stored within the vesicle, but was unavailable [173].

### 3.1. Cell Signaling

Receptor-mediated binding of EVs or of EV-derived soluble ligands to recipient cells could promote a downstream signaling cascade and elicit a pleiotropic response [93,174,175,176,177]. In these instances, it is not the intraluminal cargo that is important for intercellular communication, but surface proteins and receptors, such as fibronectin associated with fibroblast exosomes and myeloma cell exosomes.

Fibronectin-mediated binding of these exosomes to integrin on fibroblast cells and to HSPG on myeloma cells, respectively, activate p38 and pERK signaling; expression of downstream target genes, DKK1 and MMP-9, in myeloma cells; and activation of invasion-associated signaling pathways involving focal adhesion kinase (FAK) and Src family kinases (SFKs) in fibroblasts [103,120]. Another example of exosomes activating cell surface receptors is through the HSP70/TLR4 communication axis. Here, HSP70 on plasma exosomes interact with toll-like receptor (TLR) 4 on cardiomyocytes, resulting in the activation of ERK1/2 and p38 MAPK, in addition to subsequent HSP27 phosphorylation [178]. Furthermore, adding Jagged1-containing exosomes released from mesenchymal stem cells to a culture of endothelial cells triggered transcriptional changes in Notch target genes and induced angiogenesis in an in vitro model of capillary-like tube formation [179].

### 3.2. Fusion

Another possible entry mechanism is via direct fusion of the EV membrane with the cell plasma membrane and subsequent transfer of cargo molecules to recipient cells. Several factors may determine the likelihood of EV–cell fusion occurring. First, the broad consensus is that before membrane rearrangements occur, membranes are brought to within 10 nm of one another by surrogate cell-adhesion machineries. However, to overcome the energy barrier to bring them close enough (<10 nm), specialized proteins (i.e., fusogens, ligand–receptors, or glycoproteins) are required to initiate the biophysical fusion pathway. Second, while the search is on for proteins that initiate and drive cell–cell fusion, the propensity of bilayers that form fusion intermediates also depends on membrane lipids [180,181,182]. It is anticipated that the initial interactions of EVs require specific, high-affinity binding of at least two surface proteins—one protruding from the EVs, the other from the plasma membranes of the target cells. Several protein families might participate in the fusion process, including soluble NSF attachment protein receptors (SNAREs), Rab proteins, and Sec1/Munc-18-related proteins (SM-proteins) [183,184].

Supporting evidence for this delivery route can be observed in a variety of ways, including fluorescent lipid dequenching (R18) [165,166,185,186]. When this method was used to demonstrate fusion of R18-labelled exosomes with the plasma membrane of bone marrow-derived dendritic cells, confocal microscopy showed that R18 specific-labelled exosomes fuse with the plasma membrane. Furthermore, the miRNAs from these exosomes were released into the cytosol of recipient cells, a process which was measured using a luciferase activity assay [165]. Similarly, the dequenching method revealed lipid mixing of melanoma-derived exosomes and recipient cells. Moreover, in acidic extracellular conditions—as is seen in tumor microenvironments—it was shown that R18 fluorescence increased [166]. Using chemical reagents, such as botulinum toxin serotype A (BONT/A), which is used to impair SNARE-mediated membrane fusion, Ling et al. revealed that doxorubicin-loaded exosomes could be internalized to breast cancer cells via fusion [185]. Another observation of exosome-cell fusion that was published in 2017 indicated that fluorescently labeled keranocyte-derived exosomes fuse to the cell surface of stromal fibroblasts [186].

### 3.3. Endocytic Pathways

#### 3.3.1. Clathrin-Mediated Endocytosis

Clathrin-mediated endocytosis, which is the best molecularly defined endocytic pathway, is a receptor-mediated endocytic process that involves interactions between ligands on the EV surface and specific receptors present on the plasma membrane for internalization. CME regulates nutrient uptake, plasma membrane composition, signal transduction, and cell-to-cell communications through a selective uptake of cargoes, including many surface receptors [187]. Individual, internalized cargoes, packed in 60–120 nanometer-sized, clathrin-coated vesicles are triggered by the recruitment of AP2—an adaptor protein complex—and clathrin [188,189,190]. Finally, the budding off of vesicles requires dynamin. Thereafter, subsequent intracellular vesicles undergo clathrin uncoating and the internalized vesicles then enter endosomal trafficking routes from which its cargo can be returned to the cell surface or targeted to lysosomes for degradation [191,192].

Treatments affecting the formation of clathrin-coated pits can impede the entry of EVs. Chlorpromazine, a classic chemical inhibitor which prevents coated pit assembly at the cell surface [190,192,193], inhibited uptake and internalization of exosomes into cardiomycytes [194], immune cells [195,196], primary placental fibroblasts and human uterine microvascular endothelial cells [197], and hepatocyte; this led to an abrogation of exosomal cargo delivery into recipient cells and the dissemination of viruses [153]. PitStop2 (PS2)—a cell-permeable clathrin inhibitor which directly and selectively binds to the clathrin terminal domain at a site that overlaps with that used by clathrin box-containing accessory protein ligands [198]—also reduced the uptake of exosomes into various recipient cells, thus indicating that exosomal internalization is achievable via clathrin-mediated endocytosis [59,199,200,201,202].

Altering the dynamin function with dynasore, a dynamin inhibitor [203], further demonstrated the clathrin-mediated endocytosis of EVs [192,204] and macrophage-exosomes into placental trophoblasts, which induced the release of proinflammatory cytokines by the placenta [201]. To avoid problems associated with low selectivity of chemical inhibitors, genetic approaches have also been applied to inhibit exosomal endocytosis [100,154,205]. Thus, altering the expression of clathrin heavy chain with siRNA in vitro and in vivo blocked the uptake of exosomes into macrophages [206]. However, no noticeable difference in exosomal internalization into HeLa cells was observed as a result of siRNA depletion of the AP2 subunit, which is essential for the anchorage of cargo at the plasma membrane and subsequent recruitment of clathrin; this in turn allows the internalization process to proceed [207]. In a similar fashion, it has been suggested that the intercellular transfer of exosomal wild-type epidermal growth factor receptor (EGFR) into lung cancer cells is mediated by clathrin endocytosis [208]. Additionally, potassium depletion employed to assess the involvement of clathrin-mediated endocytosis resulted in dramatically reduced cellular uptake of aspirin-loaded exosomes [209].

#### 3.3.2. Macropinocytosis

In many cell types, this process supports nutrient acquisition in a regulated manner through the induction of growth factor receptors or other soluble stimuli, but this can also occur constitutively in other cell types [210]. Nakase et al. demonstrated active cellular uptake of exosomes through the macropinocytosis pathway, a process which is induced by stimulation of macropinocytosis-related receptors (EGFR and CXCR4) and oncogenic Ras proteins [211].

During macropinocytosis, a cell extends large membrane ruffles that fold back onto the cell surface and fuse to form pockets that pinch off from the plasma membrane, resulting in large intracellular vacuoles (macropinosomes) that are >250 nm in diameter [191]. Macropinocytosis involves the nonspecific uptake of extracellular materials via membrane protrusions that are driven by actin polymerization, which is impaired by F-actin-disrupting agents such as cytochalasin D [190,212]. In contrast to phagocytosis, macropinocytosis can be receptor-independent, account for fluid uptake instead of large solid particles, and occurs in almost all cell types [184,212]. Additionally, macropinocytosis is regulated by a series of small Rho-family GTPases including Rac1, Rac2, and Cdc42, which promote Arp2/3-dependent branched actin polymerization. GTPases Rab5 and Rab34 have been shown to be involved in the early stages of macropinosome formation, facilitating their fusion with early endosomes (EEs). During the macropinosome maturation, a switch from Rab5 to Rab7 function facilitates macropinosomal fusion with late endosomal/lysosomal compartments [190,212,213].

Na^+^/H^+^ exchanger activity is required to attain a critical H^+^ concentration in the immediate vicinity of the plasma membrane, which promotes actin polymerization during macropinocytosis [214]. Amiloride and EIPA (an amiloride analog) inhibit macropinocytosis by blocking this activity, thus lowering membrane pH and preventing the signaling of Rac1 and Cdc42, which are highly sensitive to pH [190,215]. In addition, phosphatidylinositol 3-kinases (PI3Ks) have been implicated in promoting macropinocytosis [213,216,217]. PI3Ks participate in a signaling cascade that stimulates membrane ruffling and macropinosome closure during macropinocytosis.

A growing list of researchers have reported the involvement of macropinocytosis in exosomal uptake into a variety of cell types, including tumor cells [160,218,219] through the use of a number of pharmacological inhibitors, including amiloride and its analogs [35,49,59,205,220,221,222,223], in addition to PI3K inhibitors such as LY294002 [202,205,224] and wortmannin [220]. Treatment of mouse hepatic stellate cells with EIPA and LY294002 showed a reduction in exosomal uptake by recipient cells. This highlighted the role of Na^+^/H^+^ exchanger- and PI3K-dependent macropinocytosis in exosomal uptake [100]. Treatment of recipient cells with lysosomal acid lipase inhibitor, lalistat 2, and lysosomotropic agents, bafilomycin A and chloroquine, resulted in reduced uptake of exosomes [100].

The role of macropinocytosis-mediated trophoblast exosome uptake by placenta cells was initially explained by using EIPA [197]. Subsequent experiments determined the signaling pathways involved in exosomal uptake by using specific pharmacological inhibitors against the PI3K-PLC-PKC-Rab34 signaling pathway, which is involved in macropinocytosis [212]. Inhibition of PI3K by wortmannin, PLC by U73122, and PKC by bisindolylmaleimide showed reduced trophoblast-derived exosome internalization by placental cells. Furthermore, siRNA-based manipulation was performed to deplete Rac1 and Cdc42, which attenuated exosomal entry into the recipient cells [197]. The role of Cdc42 was also confirmed using its pharmacological inhibitor, ML141 [197]. Contrarily, another study using EIPA, in addition to siRNA to target actin-regulating proteins (e.g., PAK1, Rac1, and Cdc42), found an insignificant reduction in exosomal uptake in HeLa cells [207].

Along with actin, microtubules are involved in macropinocytosis [210], as disruption of the polymerization of microtubules with DMA resulted in an appreciable reduction in the uptake of macrophage-derived exosomes into vascular smooth muscle cells [225]. The inhibition of the macropinocytosis pathway during chemotherapy prevented pro-tumorigenic exosomal cross talk, which suggests a potential therapeutic target for improving outcomes in ovarian cancer patients, preserving distant organs, and tumor microenvironment remodeling in pancreatic cancer [219,221,223].

#### 3.3.3. Lipid Raft-Mediated Endocytosis

Lipid rafts have been defined as small (10–200 nm), heterogeneous, more ordered domains within the membrane, enriched in cholesterol and sphingolipids, which are involved in the compartmentalization of various cellular processes, including cell signaling, membrane trafficking, and endocytosis, as well as budding for some viruses [226,227]. These rafts include proteins attached to glycosylphosphatidylinositol anchors (GPI-APs) that are inserted in the outer leaflet of membranes, proteins attached to the inner leaflet of membranes, and transmembrane proteins that have a cytoplasmic domain in addition to an outer domain that is exposed on the cell surface [228,229].

Multiple endocytic mechanisms can internalize lipid raft components or molecules that preferentially partition into rafts. Raft-dependent endocytic pathways can be classified based on their caveolin and dynamin dependence [230,231]. This includes caveolae-dependent, endocytosis-dynamin dependent- (which will be discussed further along in this article), and non-caveolae pathways- dynamin dependent or dynamin independent- including flotillin-dependent endocytosis, GTPase regulator associated with focal adhesion kinase-1 (GRAF1)-dependent endocytosis, adenosine diphosphate-ribosylation factor 6 (ARF6)-dependent endocytosis, and RhoA-dependent endocytosis [226,228,232,233].

Exosomes may also be endocytosed through lipid raft domains. The vast majority of pharmacological inhibitors of lipid raft-mediated endocytosis target cholesterol, a critical lipid constituent of lipid rafts. This includes cholesterol sequestration within the membrane by polyene antibiotics, such as filipin and nystatin [234,235,236], and extraction of cholesterol from the plasma membrane using methyl-β-cyclodextrin (MβCD) [237], which have both been used in different cancer types [111,238,239]. Lipid raft involvement in exosome internalization has been confirmed since exosomes were found to colocalize and migrate with cholera toxin B (CTXB) lipid raft markers in breast cancer and glioma cells [111,239]. Utilizing MβCD and colocalization assay with CTXB, studies have observed the involvement of lipid raft-mediated endocytosis in exosomal uptake in ovarian cancer cells and other cells. However, as caveolin-1 did not participate in exosomal uptake, it has been suggested that such uptake could occur via a caveolin-independent, cholesterol-associated pathway [70,236].

#### 3.3.4. Caveolae-Dependent Endocytosis

Caveolae-dependent endocytosis is the best characterized dynamin-dependent, clathrin-independent pathway that aids in specific cellular processes, such as plasma microdomain organization and cell signaling; cell migration and metastasis; mechano-reception and mechano-protection in certain tissues; internalization of cargos, such as virus particles, bacteria, toxins; and lipid regulation [240,241,242].

Caveolae are cholesterol- and sphingolipid-rich nanodomains on the plasma membrane that form characteristic 50–80-nanometer flask/cave-like invaginations marked by the presence of caveolin-1, an integral membrane protein [243,244]. This process involves the pinching off of vesicles by dynamin and delivery to intracellular compartments [244,245].

Expression of caveolin-1 has been described as being necessary to trigger invagination of the plasma membrane, and for the formation of morphologically defined caveolae. At the level of the endoplasmic reticulum, caveolin-2 interacts with caveolin-1 to form a high molecular-mass hetero-oligomeric complex. This complex transfers to the Golgi apparatus, binds to cholesterol, and then the caveolae precursors travel and fuse with the plasma membrane [228,242].

CDE is a highly regulated process involving the binding of various ligands to caveolin/caveolae, the clustering of cargo in caveolae to promote signal downstreaming, and tyrosin phosphorylation of caveolin resulting in caveolar internalization [242,245]. Based on ultrastructural studies, it seems likely that caveolae formation requires proteins other than caveolins, as cavin proteins (cavins 1–4) are recruited from cytosol to caveolae in the presence of caveolins and are required to stabilize the caveolae structure [240].

As caveolae are enriched in cholesterol and glycosphingolipids, a cholesterol depletion agent such as MβCD, filipin, or nystatin, among others, inhibit caveolae-dependent endocytosis which thereby inhibits EV internalization [73,105,117,219,224,246]. Caveolae-dependent uptake involves the pinching off of vesicles by dynamin. This can be prevented through the blocking of dynamin 2 activity by dynasore, which results in a significant reduction in exosomal uptake by hepatocyte, bone marrow stromal cells, and multiple myeloma cells [100,220,247]; this suggests the role of caveolae in exosomal internalization. Treatment of bone marrow stromal cells with endocytotic inhibitors suppressed multiple myeloma exosome-induced changes in multiple pro-survival and pro-proliferation pathways [247]. However, dynamin 2 is also an integral part of clathrin-mediated endocytosis. As a result, it is not possible to rule out a role for clathrin-coated vesicles in these experiments without the application of a specific knockdown or inhibition of caveolin.

Through shRNA-mediated knockdown of caveolin, some researchers have further demonstrated the role of caveolin in the internalization of tumor and non-tumor-derived exosomes [100,220,248], which subsequently attenuated the reduction in chemosensitivity to bortezomib in multiple myeloma cells [220]. Caveolar budding has been shown to be regulated by kinases and phosphatases [240,242]. Thereby, genistein—a tyrosin-kinase inhibitor that induces a degradation of the actin network in plasma membranes and prevents dynamin 2 recruitment—was used to inhibit caveolae-dependent endocytosis in murine mesenchymal stem cells, vascular smooth muscle cells, and hypatocytes [100,249,250]; this confirmed the role of caveolae-dependent endocytosis in exosomal uptake.

#### 3.3.5. Phagocytosis

Similar in many ways to macropinocytosis, phagocytosis is a specialized form of endocytosis that involves the engulfment and digestion of extracellular material of >0.5µm into plasma membrane-derived vacuoles called phagosomes [251]. Importantly, phagocytosis requires receptor-mediated recognition, as it initiates a cascade of complex signaling events that spatially guide the extension of actin-driven pseudopods that mediate particle engulfment [210,252]. Phagosomes then follow a pathway of successive fusion and fission events with EEs, late endosomes (LEs), and finally lysosomes, whereby they mature into phagolysosomes [252,253]. This pathway is used by professional phagocytic cells, such as monocytes, macrophages, neutrophils, and dendritic cells. Under certain conditions, non-professional phagocytes (e.g., fibroblasts, endothelial cells, and epithelial cells) can also perform phagocytosis [190,228].

Generally, phagocytosis is employed to internalize larger particles. However, phagocytosis has shown immune clearance of particles as small as 85 nm, which suggests the role of phagocytosis in EV internalization. Several lines of evidence support the idea that exosomes are internalized via phagocytosis [254,255]. For example, double fluorescence staining was performed to confirm the macrophage phagocytosis of osteosarcoma-derived exosomes [256]. In one study, the exosomes released by leukemia cells were shown to be taken up efficiently by phagocytes, but only a few were internalized by non-phagocytic cells. Moreover, studying the fate of exosomes after internalization revealed that almost all gold-labeled exosomes entered biospheres containing phagosomes [161].

Actin is a major player in regulating phagocytosis [253]; thus, disrupting the actin coat surrounding the phagosome prevents the formation of these structures [190,257]. Exosomal uptake was blocked by treating hepatocellular carcinoma cells with cytochalasin D and human dendritic cells with latrunculins, thus indicating that exosomes were most likely internalized via the phagocytotic pathway [258,259]. Additionally, phosphoinositides play a critical role in phgolysosome formation through the phosphorylation of the phosphoinositide- inositol headgroup by their specific lipid kinases, which generate different phosphoinositide variants that regulate the actin cytoskeleton, signaling transduction and membrane fusion/fission [253,260]. Further, treatment of cells with wortmannin and LY294002 reduced EV uptake into recipient cells [261,262]. PI3K inhibitors resulted in the dose-dependent inhibition of the internalization of leukemia exosomes by macrophages, which suggests the necessity of PI3Ks in exosomal uptake and hence the phagocytosis-dependent uptake of exosomes into cells [161]. Exosomes and other EVs secreted by malignant cells also expose PS at the outer leaflet [148], which can bind to a variety of surface receptors (e.g., integrin and TIM family members) on the cells and trigger/facilitate their uptake by phagocytosis [154,156,263,264]. Blocking TIM receptors with antibodies significantly reduced the uptake of leukemia exosomes into macrophages through phagocytosis [161].

## 4. The Fate of EVs after Internalization

The final step for EV uptake is the delivery of content to acceptor cells. Despite current knowledge about the biogenesis and uptake of EVs, little is known about the fate of endocytosed EVs [265] and their cargo, which is crucial to revealing the functional consequences of EV-mediated cargo transfers [266,267]. Once internalized, all material entering a cell converges upon a single organelle—the early endosome (EE), which is also known as the sorting endosome (SE) [172,268]. Subsequently, housekeeping proteins and receptors are recycled back to the plasma membrane directly or indirectly via a recycling endosome, or can be directed either toward lysosomes, where they enter a degradative pathway, passing through MVBs and LEs, or toward the trans-Golgi network (TGN). This is a simplified view of trafficking along the endocytic pathway, which in reality involves a complex network of structures [188,269].

Several studies have indicated lysosomes as a possible termination point following the internalization of EVs into the recipient cells [207,270,271]. Colocalization analysis verified that the transport of exosomes into pheochromocytoma and HeLa cervical cancer cells were targeted to lysosomes [207,272]. Such an endosomal-lysosomal degradative pathway may result in EV clearance and thus the escape of EV content from endosomal-lysosomal confinement would be a requirement for EV content functionality [172]. However, it cannot be ruled out that the release of partially degraded material from ruptured endosomal or lysosomal compartments could be providing a relevant source of metabolites to recipient cells [172,267].

Li H. et al. have suggested that exosomal miR-517a is capable of reaching RNA-induced silencing complex (RISC) proteins in P-bodies—a key site for cytoplasmic RNA regulation—following the internalization of trophoblast-derived exosomes into an early endocytic compartment and lysosome. The data provide support for the endocytic escape of exosomal miRNA to P-bodies [197]. Possible mechanisms for the cargo release of EVs from endosomes include: (1) membrane fusion between EVs and an endosomal membrane [66,273,274], (2) endosomal lysis, and (3) endosomal permeabilization. These mechanisms are similarly used by some viruses. This cargo escape may occur in EEs, LEs, or in the lysosome [275,276].

Early endosomes have pleomorphic structures which are identified by the association of several proteins on their cytosolic surface, including the Ras-associated protein, Rab5—which is the most well-studied molecule required for the biogenesis of EEs—along with its effector, VPS34/p150, a PI3K complex on a vesicle that promotes maturation [277,278]. To the best of our knowledge, there is little evidence in the literature for fusion with an EE to deliver EV cargo into the cytosol.

Current thinking regarding the exact process by which an EE matures toward becoming LEs is still controversial [268,279]. Maturation of early-to-LEs depends on the formation of a hybrid Rab5/Rab7 endosome, wherein Rab7 is recruited to an EE by Rab5-GTP, and LEs undergo homotypic fusion reactions, grow in size, and acquire more intraluminal vesicles [188]; alternately, it may be that specific parts of an EE could bud off and fuse with LEs [277,280]. Extracellular vesicles can discharge into the cytoplasm milieu upon fusion with a LE before reaching the lysosome [153,154,276,281].

Not long ago, dequenching of the R18 probe at the level of LEs/MVBs revealed membrane interaction between exosomes and LEs/MVBs, which is indicative of cargo release at the level of LEs/MVBs. This fusion is facilitated by lysobisphosphatidic acid (LBPA), an anionic lipid closely related to endosomal penetration of viruses [154]. Additional evidence for the fusion of exosomes in LEs comes from a paper by Joshi et al., which cites the use of exosome-expressed GFP-CD63 and HEK293T cells, which were engineered to express an anti-GFP nanobody fused to mCherry (anti-GFP fluobody) in the cytosol. They speculated that, upon fusion of the GFP-CD63 exosomes with the endosomal membrane, the GFP that was present inside the exosomes would become exposed to the cytoplasm. Consequently, the cytosolic anti-GFP fluobody would identify such a fusion event. After the team applied exosomes to the cells, mCherry punctae were formed, designating the cytosolic exposure of the exosomal cargo. The use of correlative light and electron microscopy (CLEM) revealed the presence of LEs/MVBs and lysosomes at the intracellular sites of colocalization between the exosomes and the fluobody punctae. This implied that exosomal cargo is released from LEs and lysosomes [276]. Finally, the cargo release into the cytosol was dependent on endosomal acidification and cholesterol accumulation, as the acidic pH in the LE/lysosome microenvironment favored such fusion [276,282]. Taken together, EV entry by endocytosis and subsequent cargo release via membrane fusion suggest that EVs exploit mechanisms akin to certain viruses.

Similarly to the hijacking mechanisms that are used by viruses [283], exosomes have been shown to hijack the endosomal secretory machinery of the cells that internalized them to achieve a longer distance of action and a potentially higher pathogenicity [284]. The cumulative evidence from super-resolution and electron microscopy data demonstrated that a recipient neuron can receive exosomes that were either generated by an interconnected neuron (axons extend in close proximity), or were passed on via this interconnected neuron after the processing of exogenous exosomes that have been internalized to form another neuron. High magnification images of axonal endosomes have revealed a complex structure with a mixture of endogenous and exogenous intraluminal nanovesicles, which support the notion that endosomes containing endogenous intraluminal nanovesicles fuse with the endosome loaded with internalized exogenous exosomes, which are presumably secreted [284]. Notably, the transcytosis through a cellular barrier, such as the BBB, may allow breast cancer exosomes and/or their specific content to reach distinct cells to modulate the microenvironment in the premetastatic niche to facilitate future metastasis. High spatiotemporal resolution microscopy demonstrated that endothelial recycling endocytic pathway (Rab11+ recycling endosomes) is involved in this transcellular transport [285].

Another group has described a new physiologic function of filopodia, describing it as highways for the cellular entry of exosomes and the possible site of cargo release, which are likely only hijacked by viruses and other pathogens [271]. At the base of filopodia, exosomes encapsulated into larger endocytic vesicles and were shuttled onward to scan the endoplasmic reticulum before being sorted into the lysosome as their final intracellular destination. Noteworthy is the fact that siRNA, as well as miRNA loading into RISC, binds to target mRNA, and mRNA slicing/silencing are nucleated at the rough ER membrane [286] where a directed transport of exosomes to the ER membrane would therefore allow for the efficient entry of exosomal miRNA and mRNA cargo into the RNAi and translation machineries. Whether and how exosomal cargo may be released will be important questions for future studies to investigate [271].

Recent findings have uncovered a new cellular pathway used by EVs. Analysis of the transcriptome of mesenchymal stromal cells exposed to melanoma cell-derived exosomes revealed that the regulation of eleven genes, notably those involved in inflammation, relies on the nuclear translocation of exosome-derived biomaterials [287]. After internalization, Rab7+ LEs containing exosomes and nuclear envelope invaginations come together to create a sub-nuclear compartment, where biomaterials associated with exosomes are delivered by fusion with a late endosomal membrane mediated by importin and the low pH in the LE [266,287]. The inhibition of nuclear import pathways abrogated the nuclear localization of exosome-derived biomaterials, while the inhibition of nuclear export pathways led to their accumulation within the nucleus, suggesting that their translocation is dependent on nuclear pores [287]. Earlier, Jolene et al. showed for the first time the transfer of androgen receptor (AR) and its mutant form, ARv7, via exosomes derived from prostate cancer cells to the nucleus of indolent cells devoid of AR expression following treatment with exosomes. In the absence of androgen, the transported AR activated the transcription and translation of the different genes in the targeted cells and enhanced the proliferation of acceptor cells, which may help to explain the transformation of the prostate cancer tumor from hormone responsive to hormone refractory [288]. However, the researchers did not decode the mechanism by which exosomes achieve the nuclear translocation.

## 5. Conclusions and Future Directions

This review has principally summarized the underlying mechanisms involved in exosomal targeting and recognition. In many cases, it has been demonstrated that integrins, tetraspanins, and proteoglycans help in the targeting of exosomes to certain organs and cells in vivo and in vitro. We have highlighted the mechanisms by which exosomes are internalized by cells. Endocytosis appears to be the primary method of entry by exosomes, where clathrin-mediated, caveolae-dependent, macropinocytotic, phagocytotic, and lipid raft-mediated uptake have been variously described as being prevalent. Although still not fully understood, it has been established that this mechanism mediates the internalization and transfer of EV cargoes, and depends on recipient cell type and exosomal origin. We speculate that it also depends on the proteins and lipid interactions on the exosomal membrane and cell surface, and is linked to the downstream effect and processes instigated by these vesicles. While exosomal uptake leads to the delivery of nucleic acids and proteins, internalization is not always necessary to elicit a phenotypic response. Receptor–ligand interactions that take place on the cell surface may be sufficient.

A considerable number of physiological and pathological processes are undoubtedly governed or, at least, modulated by the intervention of exosomes. They are found to maintain bodily homeostasis and are also involved in humoral immunity. Exosomes have also been shown to contribute to cancer development during each stage of the process. In addition to the effects in local tumor microenvironments, exosomes released from tumors have been shown to mediate distant cell-cell communication processes, which result in the setup of pro-tumorigenic microenvironments supportive of metastatic dissemination. This places exosomes in a privileged position to be drawn upon as a useful bank of biomarkers for a variety of tumor types and used as a non-invasive diagnostic component in medical practice. As their potential is optimized, they could become an invaluable tool in clinical therapeutic applications. Further exploration and targeting of the molecular mechanism of exosomal docking and internalization into cancer cells may inhibit the protumorigenic effect of exosomes and hence improve the patient prognoses. Alternatively, blocking exosomal cargo release by manipulating the exosome-endosome membrane fusion process or nuclear import may assist in preventing or decelerating cancer pathogenesis.

Recently, the use of tailored exosomes has shown their value as natural nanocarriers capable of targeting cancer cells with excellent biocompatibility and bioavailability. However, despite groundbreaking improvements, especially in the last decade, a number of limitations and challenges remain in regards to transforming exosome applications into clinical therapies to treat cancer. If opportunities exist to selectively deliver chemotherapeutic drugs into tumor cells via exosomes, we must improve our understating of the different gateways used by exosomes to enter cells and identify the molecules that are used by exosomes to enter cancer cells, and the endosomal escape mechanism. These molecules could be transplanted to synthetic nanodelivery tools, which theoretically may allow for the development of highly efficient, selective delivery therapeutic agents to cancer cells. This could represent a significant medical breakthrough as it may substantially minimize cytotoxic effects on healthy tissues. Excitingly, the research into exosomes is continually expanding and advancing, and there is great hope that in the foreseeable future these explorations can achieve deeper insights into their uptake pathways.

## Data Availability

Not applicable.

## Supplementary Materials

The following supporting information can be downloaded at: https://www.mdpi.com/article/10.3390/cells11091375/s1, Table S1: Targeting proteins that block exosomal interactions with and uptake by recipient cells.
