# Supplementary material for: Insight into Extracellular Vesicle-Cell Communication: From Cell Recognition to Intracellular Fate
Source: Cells. 2022 Apr 19;11(9):1375. doi: 10.3390/cells11091375 (PMC9101098; doi:10.3390/cells11091375)
Supplement: Supplementary file 1 [file cells-11-01375-s001.zip › cells-1646839-supplementary.pdf]

Supplementary Table S1.
Targeting proteins that block
exosomal interactions with and uptake by recipient cells

Target	Exosome origin	Treatment	Recipient cells
Exosomal CD9	Human perivascular stem cells[82], fibroblast cells[83]	Antibodies	Bone marrow mesenchymal stem cells[82], NUGC-3 cells, and OCUM-12 cells[83]
Cellular CD81/CD29 complex	Mesenchymal stem cells (MSCs)	Knockdown	MSCs [85]
Exosomal CD63	Brain endothelial cells	Antibodies	Brain endothelial cells[86]
Exosomal CD151/TSPAN8	Rat pancreatic adenocarcinoma	Knockdown	Stroma cells and hematopoietic cells [88]
Exosomal integrin αV and β3	Leukemia	Antibodies	Human choroid plexus papilloma cells[94]
Exosomal integrins	Human primary astrocytes	RGD peptides	Neurons[95]
Exosomal integrin $\alpha2\beta1$	Cancer-associated fibroblasts (CAFs)	TC-I 15 (an $\alpha2\beta1$ integrin inhibitor)	Lung fibroblasts[96]
Exosomal $\alpha3\beta1$ integrin	Ovarian cancer	Cyclic nonapeptide, LXY30	Ovarian cancer[98]
Cellular CD46, integrin α5, and integrin αV	Brain-metastatic cancer cell line	Neutralizing antibodies against integrin α5 and integrin αV, and siRNA for CD46	Blood-brain barrier endothelial cells (hCMEC/D3 cells) [72]
Exosomal LFA-1	Macrophages	Antibodies	Brain microvascular endothelial cells (BMECs)[73]
Cellular integrin	Hepatocytes	Echistatin, a potent inhibitor of RGD-binding integrin	Hepatocytes and hepatic stellate cells [100]
Exosomal laminin γ2	Metastatic oral squamous carcinoma cells	Knockdown	Lymphatic endothelial cells[102]
Cellular integrin ITGB3	Breast cancer	Knockdown	Breast cancer [101]
Cellular integrin	Human embryonic kidney cells	RGD peptide	Retinal cells [105]
Cellular integrin α6 and αX	Gastric epithelial cells	Knockdown	Gastric cancer cells[59]
Cellular heparan sulfate PGs (HSPGs)	Neural stem cells (NSCs)	Heparinase III	Brain endothelial cells[110]
Cellular HSPG	Glioma cells	HS mimetic heparin; HS lyases; false substrate, 4-itrophenyl β-D-	Glioma cells[111]

		xylopyranoside;<br>and NaClO3	
Cellular HSPGs	Glioma cells	Heparin, HS lyase,<br>and heparinase	Glioma cells[112]
Cellular<br>and exosomal HSPG	Oral squamous cell<br>carcinoma[113],<br>bladder cancer [116]	Heparin	Oral squamous cell<br>carcinoma[113],<br>bladder cancer[116]
Cellular surface<br>syndecan-4 (SDC4)	Breast cancer	Knockdown	Glioblastoma cell line[118]
Exosomal<br>and cellular HSPG	Myeloma cell	Heparitinase	Myeloma cells[120]
Exosomal<br>terminal sialylation	Glioblastoma	Neuraminidase (NA)	Dendritic cells[130]
Exosomal N- glycan	Melanoma cells	Peptide: N-glycosidase F<br>(PNGase F)	Peritoneal<br>macrophages[131]
Exosomal<br>terminal sialylation	Prostate cancer	NA	Macrophage cells[135]
Exosomal<br>terminal sialylation	Liver progenitor cells	NA	Lungs and<br>axillary lymph nodes[136]
Cellular siglec-3	MSCs	Anti-CD33 (siglec-3)<br>antibody	HeLa cells[128]
Exosomal glactin-5	Reticulocyte	Purified galectin-5	Macrophages[142]
Cellular LFA-1<br>and DEC205	Dendritic cells	Antibodies	Dendritic cells[143]
Cellular DC-SIGN	Breast milk	Antibodies	Monocyte-derived<br>dendritic cells[144]
Cellular CD169	B cells	Knockdown	Macrophages[145]
